# Ruminant Fecal Contamination of Drinking Water Introduced Post-Collection in Rural Kenyan Households

**DOI:** 10.3390/ijerph17020608

**Published:** 2020-01-17

**Authors:** Latifah Hamzah, Alexandria B. Boehm, Jennifer Davis, Amy J. Pickering, Marlene Wolfe, Maryanne Mureithi, Angela Harris

**Affiliations:** 1 Department of Civil and Environmental Engineering, Stanford University, Stanford, CA 94305, USA; latifah@stanford.edu (L.H.); aboehm@stanford.edu (A.B.B.); jennadavis@stanford.edu (J.D.); marlene.wolfe@stanford.edu (M.W.); 2 Woods Institute for the Environment, Stanford University, Stanford, CA 94305, USA; 3 Department of Civil and Environmental Engineering, Tufts University, Medford, MA 01255, USA; amy.pickering@tufts.edu; 4 Innovations for Poverty Action, Nairobi, Kenya; maw206@gmail.com; 5 Department of Civil, Construction, and Environmental Engineering, NC State University, Raleigh, NC 27695, USA

**Keywords:** microbial source tracking, fuzzy set qualitative comparative analysis, stored water, source water, fecal bacteria, ruminant contamination

## Abstract

In sub-Saharan Africa, many families travel to collect water and store it in their homes for daily use, presenting an opportunity for the introduction of fecal contamination. One stored and one source water sample were each collected from 45 households in rural Kenya. All 90 samples were analyzed for fecal indicator bacteria (*E. coli* and enterococci) and species-specific contamination using molecular microbial source tracking assays. Human (HF183), avian (GFD), and ruminant (BacR) contamination were detected in 52, two, and four samples, respectively. Stored water samples had elevated enterococci concentrations (*p* < 0.01, Wilcoxon matched pairs test) and more frequent BacR detection (89% versus 27%, *p* < 0.01, McNemar’s exact test) relative to source water samples. fsQCA (fuzzy set qualitative comparative analysis) was conducted on the subset of households with no source water BacR contamination to highlight combinations of factors associated with the introduction of BacR contamination to stored water supplies. Three combinations were identified: (i) ruminants in the compound, safe water extraction methods, and long storage time, (ii) ruminants, unsafe water extraction methods, and no soap at the household handwashing station, and (iii) long storage time and no soap. This suggests that multiple pathways contribute to the transmission of ruminant fecal contamination in this context, which would have been missed if data were analyzed using standard regression techniques.

## 1. Introduction

Access to safely-managed drinking water services has been steadily improving throughout the world [1]. The World Health Organization (WHO)/United Nations Children’s Fund (UNICEF) Joint Monitoring Program (JMP) for Water, Sanitation and Hygiene (WASH) defines a safely-managed drinking water service as one that is located on premises, available when needed and free from contamination [2]. Still, in 2015, 2.1 billion people, or approximately 29% of the global population, did not have access to a safely-managed drinking water service, including 76% of the population in sub-Saharan Africa [2]. The majority there (50% of the population) must travel to collect drinking water from a communal but improved source, storing it in their homes for daily use. The remainder (26% of the population) use unimproved sources like surface water or unprotected springs [2].

In the cases where water is stored in the household for later use, drinking water can become contaminated after collection from the source, resulting in a significant decline in quality [3,4,5]. Contamination can be introduced to stored water by hands or fomites entering the water [3,6,7]. Contamination of stored water is more severe when storage vessels are earthenware containers [4] or left uncovered [3], although safe storage vessels with a narrow mouth, lid, and tap have been shown to reduce contamination and improve health outcomes [8]. Point of use (as opposed to point of delivery) water treatment methods have been suggested to protect the quality of water consumed; however, long-term and reliable use of these types of treatment methods is needed to protect health [9]. Overall, there is a lack of understanding of how various household attributes and human behaviors affect stored water quality. Although studies in Tanzania [10], Peru [11], and Bangladesh [12] all reported contamination of stored drinking water supplies, none identified pathways of contamination that would describe a majority of contamination events. In addition, a recent study of stored water quality in rural and peri-urban Tanzania [13] explored how various factors (e.g., human behavior and household and water characteristics) were associated with stored water quality. However, the resultant models could only explain a small amount of variance in the water quality measurements [13], comparable to the models used in other studies. In the present study, we apply a qualitative modelling approach, fuzzy set qualitative comparative analysis (fsQCA), to explore how various combinations of household and environmental attributes combined with human behaviors are associated with stored water contamination. This will provide additional insight into the complexities of fecal contamination transmission in household settings, particularly if contamination is being transmitted via multiple pathways.

fsQCA has several advantages over standard regression methods [13]. For example, it does not require the analyst to specify the interactions between the causal conditions *a priori*. Therefore, fsQCA does not rely on the analyst’s preconceived notions about the relationships in order to identify complex interactions among the causal conditions that give rise to the outcome. It also does not assume causal symmetry, i.e., the conditions leading to the absence of the outcome are not presumed to be the opposite of those leading to its presence. fsQCA also allows for meaningful categories of values for the causal condition relative to the outcome to be coded explicitly. This removes irrelevant variation in the indicator and minimizes the influence of outlier cases in the analysis. fsQCA has been used to identify complex relationships in renewable energy [14], online shopping and marketing [15], organizational performance [16], and, recently, WASH, where it was used to study combinations of community, program, and external conditions associated with the long-term sustainability of rural water supply programs [17]. The present study is a unique, but fitting, application of fsQCA to explore combinations of factors associated with the introduction of fecal contamination to environmental water samples.

Globally, microbial guidelines for drinking water quality are based on concentrations of FIB (fecal indicator bacteria) including *E. coli*, enterococci, and total and fecal coliforms [18]. FIB are used as water quality indicators because they are present in high concentrations in sewage and feces [19] and are relatively inexpensive to measure compared to pathogens. Conceptually, FIB concentrations should be high when fecal pathogens are present and thus their high concentrations in drinking water should indicate that exposure to fecal pathogens is likely. Some studies have found positive associations between FIB concentrations in drinking water and adverse health outcomes [20,21]. However, other studies have shown either a lack of correlation between FIB and pathogen presence in drinking water [22,23,24] or a lack of association between human health outcomes and FIB concentrations in drinking water [7,25,26]. Differing results among studies might suggest that the conceptual model does not consider important sources or fate processes that differentially affect FIB and pathogens in drinking water. For example, FIB can come from a variety of sources other than human feces or sewage including non-human animals, and health risks vary by fecal sources [27]. Non-human animal feces do not contain human viruses, an important etiology of waterborne illness [27]. Host-associated fecal indicators have been proposed as a means for identifying different types of animal fecal contamination in water [28,29,30]. Host-associated fecal indicators are typically detected using molecular biological methods like PCR (polymerase chain reaction). Their use to identify sources of fecal contamination is referred to as MST (microbial source tracking). While MST methods have been used within high-income countries to identify sources of environmental contamination [31], they have rarely been applied in low- and middle-income countries [6,12,32,33,34,35,36,37].

This study was conducted among households enrolled in a large-scale randomized controlled trial of water, sanitation, handwashing, and nutrition interventions in rural Kenya (WASH Benefits Kenya). The trial found that the water intervention (chlorination) improved microbial water quality as measured by *E. coli*, but none of the interventions reduced *E. coli* contamination on child hands or on sentinel toy balls [38], and none of the interventions reduced child diarrhea or improved child growth [39]. The study investigators concluded that the interventions were not able to sufficiently reduce fecal contamination in the household environment [40]; one potential explanation is that animal feces were a substantial source of fecal contamination in study households [38]. This study uses MST to investigate the animal hosts that contribute fecal contamination to stored and source water supplies collected from a subset of study households. In addition, fsQCA is used to identify combinations of causal conditions that lead to the introduction of fecal contamination to the household stored water supply.

## 2. Materials and Methods

### 2.1. Study Site and Sample Frame

Water and fecal samples were collected from a subset of households enrolled in the WASH Benefits Kenya study [39]. All the households that participated in the present study were visited during baseline data collection for the main trial between 26th August and 6th Sept 2013 [41]. Villages were eligible for enrollment if they were rural (defined as having <25% of residents living in rental houses, <2 gas/petrol stations and <10 shops) [41], relied largely on communal water sources, had unimproved sanitation facilities, and were not participating in any ongoing WASH or nutrition programs [39]. The households are located in rural areas of Kakamega county in western Kenya (0°17′01.5′′ N, 34°45′04.5′′ E, map of households previously published [42]), which are populated mainly by subsistence farmers [41].

### 2.2. Fecal Sample Collection and Processing

Fecal samples were collected to validate molecular MST targets for the study area. Fecal samples were collected from chickens (*n* = 20), cows (*n* = 20), goats (*n* = 20), dogs (*n* = 17), sheep (*n* = 20) and humans (*n* = 19), which represent the animals most common in the area [43]. The non-human fecal samples were collected from household-owned animals throughout the study areas using a sterile fecal specimen collection container with a spoon built into the cap. Field staff were trained on host identification of fecal specimens. More than 2 g of feces was collected from each pile sampled, with care taken to avoid including soil. Although efforts were made to target fresh feces that appeared to be deposited within the past day, the precise age of each specimen was unknown. Human fecal samples were collected from adult females (ages 18–45 years) and male and female children aged under 2.5 years to achieve variation in age and gender. A stool sample kit, consisting of a sterile stool collection tube with scoop, aluminum foil, and gloves, was left with the household and then collected the next day. The households which supplied fecal samples are different from those that supplied the water samples (described below). Collected samples were stored in coolers on ice and transported to a local laboratory for processing.

Aliquots of equal mass from between two to four individual fecal specimens of the same animal type were combined to form a 2.0 g composite as indicated in Table S1. Molecular grade water (Thermo Fisher Scientific, Waltham, MA, USA) was added to the composite samples to make 20 mL fecal slurries in DNA-sterile 50 mL centrifuge tubes (Fisher Scientific, Hampton, NH, USA). The concentrations of *E. coli* and enterococci were enumerated via membrane filtration of dilutions of the slurries through Millipore HABG 047 S6 0.45 μm pore size filters (Fisher Scientific) and placing them on MI and mEI selective media (EPA methods 1604 [44] and 1600 [45], respectively). Samples were processed at multiple dilutions to ensure that the number of colonies on plates was between 10 and 500 CFU [46]. In addition, 2 mL of the slurry was membrane filtered through 0.4 μm pore size polycarbonate filters (Isopore Millipore Filter, Fisher Scientific) for molecular analysis. The filter was treated with 0.5 mL of RNAlater solution and allowed to sit atop the filter for 5 min before it was vacuum filtered through. The filters were then stored in microcentrifuge tubes with glass beads (Generite, North Brunswick, NJ, USA) at −20 °C until transport to a US-based laboratory (within 1 month). Filters of avian fecal samples were heat treated (after treatment with RNAlater) at 74 °C for 30 min prior to transport according to United States Department of Agriculture regulations. Samples were then stored at −80 °C until DNA extractions were performed in 2014. One fecal composite per animal source type was processed in duplicate to assess intrinsic assay variability. Lab process blanks were created each field sampling day using molecular grade water (i.e., diluent of the slurries).

### 2.3. Household Water and Survey Data Collection 

Locally trained enumerators visited each of 45 study households to interview the primary female caregiver in Kiswahili and make observations about WASH behaviors and infrastructure in the household. All respondents provided free and informed consent to participate. Enumerators recorded observed household water sources, sanitation facilities, hand washing facilities, and animal presence within the home and compound. Enumerators also collected self-reported handwashing behaviors, water treatment and extraction methods, household building material, and drinking water storage time from the respondent. All baseline survey data were recorded using a netbook laptop, with the survey programmed in Blaise (Westat USA, Rockville, MD, USA). The study was conducted in accordance with the declaration of Helsinki, with the study protocol approved by the Committee for the Protection of Human Subjects at the University of California, Berkeley (protocol number 2011-09-3654), the institutional review board at Stanford University (IRB-23310), and the scientific and ethics review unit at the Kenya Medical Research Institute (protocol number SSC-2271) [39].

At the same time as the household survey, enumerators collected stored drinking water samples from the households. The enumerator asked the respondent to collect water as they normally would for drinking and deposit the water sample directly into a sterile Whirl-pak (Nasco, Fort Atkinson, WI, USA) sample bag (approximately 500 mL volume). On the same day as the household survey, a separate team of enumerators collected a water sample directly from the source where the stored water had been collected (as reported by the respondent) and recorded the water source type (i.e., borewell, shallow well, spring, or piped water). After collection, water samples were stored in a cooler on ice, transported to a local lab, and processed within 12 h of collection. *E. coli* and enterococci were enumerated in 100 mL volumes of the water samples using membrane filtration with 0.45 μm pore size HA filters (Millipore, Burlington, MA, USA) on MI and mEI selective media (EPA standard methods 1604 [44] and 1600 [45], respectively). Results are presented as concentrations in units of CFU (colony forming units) per 100 mL. If the plate count was too numerous to count (i.e., greater than ~500 CFU), then 500 CFU/100 mL was used as a substitution for the counts. In addition, 100 mL volumes of the water samples were membrane filtered, transported, and stored using the same protocol as with the fecal samples. DNA extractions were performed in 2018. Lab process blanks were processed each field sampling day using molecular grade water.

### 2.4. Molecular Processing of Fecal and Environmental Samples

In the US-based laboratory, DNA was extracted from the fecal sample filters using the commercial DNA EZ extraction kit (Generite). Ten to twenty samples were extracted at a time, and an extraction blank (i.e., reagents only, with no sample filter included) was created with each extraction set. The following MST Taqman qPCR (quantitative PCR) assays were performed on the fecal sample DNA extracts: HF183 taqman [47], BacHum [48], humM2 [49], BacCow [48], Rum2Bac [50], and BacR [51]. An avian SYBR green assay termed GFD was also performed [52]. Cycling parameters and primer and probe concentrations were as described in the referring manuscript of each assay. The master mixes used for the MST assays are shown in Table S2.

DNA from the water samples were extracted from their filters using the commercial DNA EZ extraction kit (Generite). Between 5 and 23 samples were extracted at a time, with an extraction blank (i.e., no sample filter included) created with each extraction set. The following qPCR assays were performed on the water sample DNA extracts: HF183 Taqman [47], BacR [51] and Avian SYBR green GFD [52] following the same protocols as for the fecal samples. These were the best performing assays for human, ruminant, and avian species, respectively, based on the validation study.

Plasmid standards required for each assay were either purchased from IDT (San Jose, CA, USA) or extracted from plasmid-carrying *E. coli* grown from existing stock using the commercial QIAprep Spin Miniprep Kit (Qiagen, Valencia, CA, USA). The concentrations of plasmid standards were quantified using Nanodrop (Thermo-Scientific, Wilmington, DE, USA). Each qPCR plate processed included a standard curve run in triplicate with concentrations of standard (Table S2) ranging from 10^1^ copies per μL of DNA extract to 10^5^ copies per μL of DNA extract. Each reaction contained 2 μL of DNA extract. Triplicate no-template controls were included with each 96-well plate. All fecal samples were processed in triplicate and all water samples were processed in duplicate.

For each assay, a master standard curve was created by combining the standard curves from individual qPCR plates. C_T_ (mean cycle threshold) values were assigned using 0.03 as the fluorescence threshold for all assays. The master standard curve was used to calculate molecular marker concentrations in samples using the sample’s C_T_. A sample was considered detected within the ROQ (range of quantification) if the sample’s mean C_T_ value corresponded to a concentration between 10^1^ copies per μL DNA extract and 10^5^ copies per μL DNA extract. If the sample had a mean C_T_ value that corresponded to fewer than 10 copies per μL DNA extract, the sample was reported as DNQ (detected but not quantifiable). If the sample had a concentration above 10^5^ copies per μL DNA extract, then the sample was decimally diluted until its concentration was within the ROQ. If the sample had an undetermined C_T_ value for both replicates, then the sample was reported as a ND (non-detect). For the fecal samples, if two out of three of the reactions were undetermined, the sample was reported as a ND. Water sample results within the ROQ were reported as the average number of molecular marker copies detected per mL of water sample; if one out of two reactions was undetermined, the sample was reported as a ND unless the other reaction was within the ROQ, in which case the sample was reported as a DNQ. A modified spike and dilute method was used for assessing inhibition in the water and fecal samples. Specific details are in Appendix A.

### 2.5. MST Validation Data Analysis 

MST assay validation was conducted using quantitative and binary methods following the approach outlined by Boehm et al. [53]. For the quantitative analyses, the chosen metric was the concentration of MST molecular marker detected in a fecal composite sample normalized by the number of enterococci colonies formed, i.e., copies per CFU ENT. For an MST assay to be labeled as sufficiently sensitive, the median concentration of the MST molecular marker in the target host feces (that is, the feces of the targeted animal host) should be greater than 10 copies per CFU ENT. Ten copies per CFU ENT represents a 100 mL environmental sample having 100 CFU ENT/100 mL if the filtered sample yielded 100 μL of eluent (i.e., 1 CFU ENT/μL of eluent) after DNA extraction and had a lowest detectable concentration of 10 copies/μL DNA extract (therefore 10 copies/CFU ENT) in the qPCR reaction. An MST assay was considered specific if the concentration of the MST marker in all non-target host feces samples were lower than the lowest concentration detected in a target host fecal sample, with only concentrations detected within the ROQ considered as described previously [53].

A binary analysis of MST assay performance was also conducted on the basis of the presence/absence of the molecular marker in the sample. A fecal sample was considered positive for an MST marker if it returned DNQ or ROQ but considered negative if it returned ND. The sensitivity::

$$Sensitivity=\;\frac{True\;Positive}{True\;Positive+False\;Negative}$$

reported as a percentage, is the fraction of positive target samples tested (i.e., true positives) over the total number of target samples processed. The specificity:

$$Specificity=\;\frac{True\;Negative}{True\;Negative+False\;Positive}$$

reported as a percentage, is the fraction of negative non-target samples identified (i.e., true negatives) over the total number of non-target samples processed. An 80% threshold was set for an assay to be labelled sensitive and/or specific [53].

### 2.6. Data Analysis—Water Samples 

Water quality indicators (i.e., MST molecular markers and FIB) were used in various analyses in binary (presence/absence) and continuous form (concentration of the molecular marker in the water sample). When continuous variables were used for FIB data, substitutions for NDs were necessary because the data were log_10_-transformed [12]. As such, NDs for FIB (*E. coli* and *enterococci*) were replaced with 0.5 CFU per 100mL water sample.

Statistical analyses included the Wilcoxon matched pair and rank sum tests and the McNemar’s exact test. The Wilcoxon matched pair and McNemar’s tests were used to assess trends in both the MST molecular markers and FIB indicators by assessing the paired source and stored water samples from households, whereas the Wilcoxon rank sum test was used to identify correlations between the MST molecular markers and FIB indicators used in the study. Wilcoxon tests were conducted because the data were not normally distributed, while the McNemar’s exact test was conducted because the sample size of households was small. When tests used binary data for the presence of MST molecular markers, a marker was considered present if it was detected in a sample (i.e., ROQ or DNQ). *p* values below 0.05 were considered statistically significant. All analyses were implemented in R.

### 2.7. fsQCA

To identify relationships between ruminant contamination in stored water and combinations of household behaviors and characteristics, an fsQCA approach was employed using the fsQCA3.0 software downloaded from fsqca.com. The household sample for the fsQCA analysis was drawn from the 45 study households for which stored and source water samples were available for FIB and MST analyses. The 33 households ultimately included in the fsQCA analysis were those with no detected ruminant contamination, i.e., BacR molecular marker, in the source water supply. This choice was made to focus on the identification of factors that are associated with post-supply introduction of contamination.

**Figure 1:**
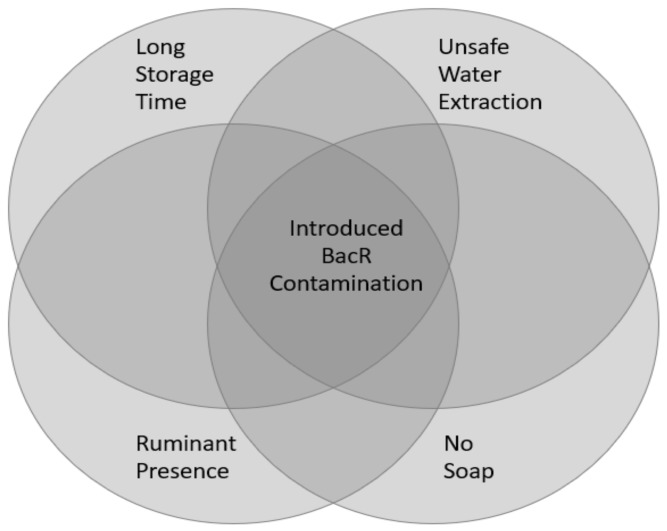
Conceptual model of the interconnected factors that are associated with the introduction of ruminant contamination to a household’s stored water supply.

The conceptual model, shown in Figure 1, shows four causal conditions that are theorized to be associated with the outcome of introduction of ruminant fecal contamination (indicated by detection of BacR) to a household’s stored water supply. The outcome and causal conditions used for the fsQCA analysis are described in Table 1, which lists each construct, its definition, its theoretical relevance to the conceptual model, its hypothesized effect on the outcome, the indicator used to measure it, and how it is scored as input to the fsQCA analysis. Indicators were chosen for their validity and reliability as proxies for the construct based on prior experience given the constraints that data had to be complete for all households and heterogenous. The indicator was considered sufficiently heterogenous if there were no more than 85% of households reporting the same value [54,55].

The causal conditions and shorthand descriptions of their indicators are: the presence of ruminants in the household’s broader compound (“Ruminants”), unsafe water extraction methods where water was obtained from the storage receptacle by dipping hands or an object as opposed to being poured (“Unsafe Extraction”), the opportunity for introduction of contamination due to long storage time in the household after collection (“Long Storage Time”), and the lack of preventative measures against the introduction of contamination by hands by having neither soap nor water at the household’s handwashing station (“No Soap”). In addition to being an indicator for the construct of prevention of contamination by hands, soap may also serve as a combined indicator of wealth and education since it could reflect both knowledge that handwashing is important and having the means to afford soap. This was demonstrated by intermediate analysis steps, with details provided in Appendix B.

To conduct the fsQCA analysis, a .csv file was created with the columns showing the causal and outcome conditions and the rows listing the values of their indicators for each included household. The indicators of the causal and outcome conditions for each case (household) were coded with values ranging between 0 and 1. A value of zero for an indicator signifies that the household is ‘fully out’ of the set of households with that characteristic, whereas a value of 1 for an indicator signifies that the household is ‘fully in’ the set of households with that characteristic [54]. A value between the two therefore suggests that a case is more ‘in’ than ‘out’ of the set if larger than 0.5 and vice versa if smaller than 0.5, with 0.5 the score of maximum ambiguity. The results of the analysis are combinations that are evaluated in terms of their consistency and coverage [54], where:

$$Consistency=\;\frac{\#\;of\;cases\;with\;both\;the\;causal\;and\;outcome\;conditions\;}{\#\;of\;cases\;with\;causal\;condition}$$

$$Coverage=\;\frac{\#\;of\;cases\;with\;both\;the\;causal\;and\;outcome\;conditions\;}{\#\;of\;cases\;with\;outcome\;condition}$$

Consistency and coverage scores above 0.8 are conventional thresholds for establishing combinations [54].

A necessary condition implies that all cases exhibiting contamination have the causal condition present. A sufficient condition is that there is contamination whenever that causal condition is present, but that there may be cases of contamination where the condition is not present.

fsQCA places an emphasis on ensuring that differences in the coded values reflect meaningful and substantive variation, with indicator values pre-processed from survey data to reflect the coding scheme described in Table 1. For example, it is less important to capture the precise number of ruminants that live in a compound than it is to group the number of ruminants into herd sizes that represent differential fecal contamination risks. However, given the lack of existing literature for what meaningful variation may be across the indicators, most causal conditions were coded as binary, i.e., as either 1 or 0. Indeed, the only construct for which the indicator was coded in a continuous manner with values along a spectrum from 0 to 1 was the opportunity for the introduction of contamination (i.e., length of time water has been stored in home). Previous literature indicates that storing water for a period of 24 h substantially increases the probability of contamination being introduced [11]. As such, “Long Storage Time” was coded as 0.95 if 24 h had elapsed since collection to represent being fully in the set of households where storage time was likely to be associated with contamination. “Long Storage Time” was coded as 0.05 if an hour had elapsed to represent being fully out of the set, and as 0.5 if 4.5 h had elapsed to represent maximum ambiguity. The fsQCA software then scored all other storage time lengths with respect to these three set data points [54] using the calibrate sub-function of the Compute function as detailed in Appendix B.

Thus, the fsQCA analysis conducted had detection of the BacR molecular marker in the household’s stored water supply as the outcome condition and “Ruminants”, “No Soap”, “Unsafe Extraction”, and “Long Storage Time” as the causal conditions hypothesized to be most associated with this outcome. Further analysis details are presented in Appendix B.

## 3. Results

### 3.1. Quality Assurance/Quality Control

Fecal composites and water samples were processed alongside 19 process blanks. All process blanks were free from contamination when subjected to FIB enumeration and the 7 MST assays. DNA extractions from the fecal composites and water samples generated five and 10 extraction blanks respectively, which were processed in duplicate and all free from contamination when subjected to the three chosen MST assays. Thirty-five inhibition tests using water and fecal DNA extracts showed no inhibition. Data from the standards were combined to generate master curves (Figure S1) with a LLOQ (lower limit of quantification) of 10 copies per mL water sample for each assay.

### 3.2. MST Validation Study

Of the seven MST assays tested, the BacR, HF183 and Avian GFD assays were found to be effective at detecting and distinguishing ruminant, human, and avian fecal contamination, respectively, in Western Kenya. All were specific in the quantitative analysis and 100% sensitive in the binary analysis, as shown in in Table 2. These MST assays were selected to analyze the water samples.

**Figure 2:**
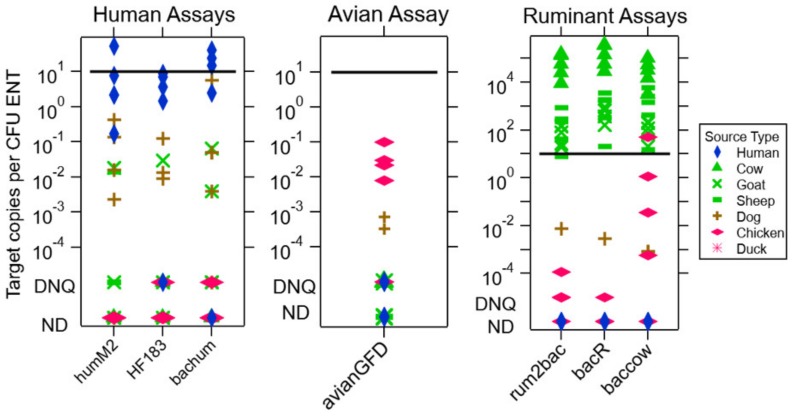
Concentrations of MST assay molecular marker copies per culturable ENT in fecal material, which represents the ratio of species-specific bacteria (target copies) to fecal indicator bacteria (CFU ENT). Higher concentrations suggest that species-specific contamination can be detected with less feces in the water sample as measured by ENT. Fecal samples are from chickens, ducks, cows, goats, sheep, dogs, and humans. Humans, ruminants (cows/goats/sheep), and avian species (chicken/ducks) are the target fecal sources for these assays. The human assays tested are humM2, HF183, and bachum, the avian assay tested is avianGFD, and the ruminant assays tested are rum2bac, bacR, and baccow. At the bottom of the molecular marker copy scale, samples that had the MST molecular marker DNQ or ND are plotted. The black line marks the sensitivity threshold of 10 copies per CFU ENT. This would represent the threshold of detection in the qPCR process for samples containing 1 CFU ENT, the smallest unit of contamination above WHO guidelines.

Of the human-associated fecal assays (HumM2, HF183, and BacHum), none met the quantitative sensitivity criterion, i.e., none had the median concentration of the MST molecular marker in human feces as being greater than 10 copies per CFU ENT (Figure 2). However, all three assays had a wide range of target detection that crossed this threshold, with 80% or more of the human fecal composite samples returning positive (i.e., were detected within the ROQ) and therefore meeting the binary sensitivity criterion (Table 2). Still, heterogeneity in the detection of the target meant that it was sometimes not detected even with a composite sample from 4 individuals, as can be seen with BacHum. The HF183 assay satisfied the quantitative specificity criterion because concentrations of the molecular marker in all non-human fecal composites were below those in the human ones, whereas this was not true of the HumM2 and BacHum assays. None of the assays met the binary specificity criterion, i.e., fewer than 80% of non-human fecal composites correctly returned a negative result. As such, HF183 was identified as the most effective of the human-associated assays tested.

Of the ruminant-associated assays, all three (Rum2Bac, BacR and BacCow) satisfied both the quantitative and binary sensitivity criteria, having a range of detection of the ruminant target spanning more than four orders of magnitude, with the lowest concentration of target around the 10 copies per CFU ENT threshold. However, only Rum2Bac and BacR met the quantitative specificity criterion and only BacR fulfilled the binary specificity criterion. As such, BacR was identified as the most effective ruminant-associated assay tested. In addition, although the Avian GFD assay did not meet either the quantitative sensitivity or binary specificity criteria, it did meet the quantitative specificity and binary sensitivity criteria and was thus deemed to have acceptable performance for use on environmental samples.

### 3.3. Household Characteristics

The 45 households from which source and stored water samples were collected had an average household size of 5, with the mother most commonly having completed primary school (Table 3). Nine percent (9%) of households had electricity, 42% owned bicycles, and 76% owned mobile phones. All households had access to a toilet facility, with 47% of households having access to private sanitation, with the remaining sharing their toilet with anywhere between one and five additional households (mean: 2.2). However, only 4% of all facilities were classified as improved sanitation based on the JMP definition; this definition requires that private latrines have a concrete slab [2] that few in our study did.

### 3.4. Animal Characteristics and Host-Associated MST Marker Results

Forty-seven percent (47%) of households self-reported that they owned ruminants, 31% dogs, and 76% chickens. There was variation in the prevalence of the MST molecular markers in the water samples across study households. Avian GFD was detected in 2% of all samples (2 of 90), HF183 in 4% (4 of 90), and BacR in 58% (52 of 90). The BacR MST molecular marker was prevalent and found in the stored and/or source water samples of 91% of households (41 of 45), or 27% of source water samples (12 of 45) and 89% of stored water samples (40 of 45). When detected, log_10_-transformed copies per 100 mL water sample of Avian GFD were between 2.1 and 6.9, HF183 were between 2.5 and 2.9, and BacR were between 2.1 and 4.6. There were no bivariate associations between the self-reported presence of animals in the compound and the detection of MST markers (see Appendix C).

### 3.5. Water Supply Characteristics

**Figure 3:**
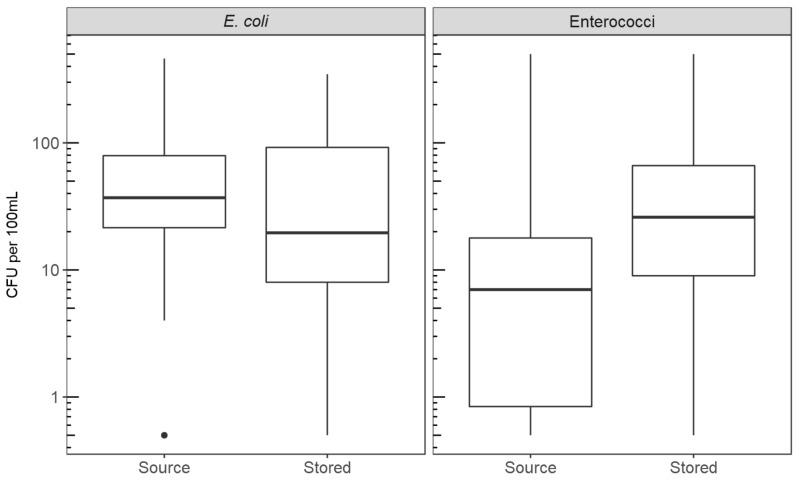
Boxplots of the concentrations (CFU/100mL) of *E. coli* (left) and *enterococci* (right) in the source and stored water samples. The midline of the box represents the median of the data, with the upper and lower bounds of the box showing the first and third quartile. The whiskers show the extremes of the data that are within 1.5 times the interquartile range. Any data points outside this range are plotted as outlier circles (one outlier for E. coli concentration in source water).

Observed household water sources included borewells (2.2%), streams (2.2%), protected dug wells (8.9%), unprotected dug wells (2.2%), protected springs (73.3%), and unprotected springs (11.1%). Springs are sources where water comes from the subsurface and is accessible at the ground level without any further technology or intervention, whereas wells are dug to groundwater. Protected springs and wells had a concrete lining, whereas unprotected versions did not. For water treatment, 13% of households self-reported that they treat their water regularly by using methods including bottled chlorine, boiling, sieving it through cloth, or using a Lifestraw filter. However, only 4% of households self-reported having treated the stored water from which a sample was taken. There was extensive contamination of both stored and source drinking water with *E. coli* and enterococci (Figure 3). Ninety-five percent of water samples (93% of source and 98% of stored) had *E. coli* detected in the 100 mL sample with a median of 33 CFU/100 mL; 85% (75% of source and 95% of stored) had enterococci detected in the 100 mL sample with a median of 11 CFU/100 mL.

### 3.6. Ruminant Contamination Introduced to Stored Water

Our study found evidence of ruminant fecal contamination and enterococci introduced into a household’s drinking water post-collection from the source. When analyzing stored and source water sample pairs matched by household, the BacR molecular marker was detected in stored water but not in source water for 64% of households, with the reverse occurring in only 2% of households. The difference was statistically significant (Table 4, McNemar’s exact test, *p* < 0.01). Similar analysis using FIB as the dependent variable showed that the stored water samples had significantly higher log_10_-transformed enterococci concentrations (Figure 3, Wilcoxon matched-pairs, *p* < 0.01) relative to the source water samples, with median values in the source and stored water samples of 7 and 26 CFU/100 mL, respectively.

There was no significant difference in concentrations of *E. coli* between source and stored water (Figure 3, Wilcoxon matched-pairs, *p* = 0.25), with median values of 37 and 20 CFU/100 mL respectively. No statistically significant difference was found in the occurrence of HF183 (Table 4, McNemar’s exact, *p* = 0.5) or Avian GFD (Table 4, McNemar’s exact, *p* = 0.5) targets between source and stored water samples. Conducting the analyses with only the subset of households with springs as their water source (the most common source) did not change the results (Appendix D).

### 3.7. Correlations Between Fecal Indicators

Across all water samples, BacR presence was positively associated with enterococci concentrations (Wilcoxon rank sum, *p* < 0.01) whereas it was not associated with *E. coli* concentrations (Wilcoxon rank sum, *p* = 0.95). Neither *E. coli* (Wilcoxon rank sum, *p* = 0.89) nor enterococci (Wilcoxon rank sum, *p* = 0.48) concentrations were associated with the presence of Avian GFD contamination. However, both *E. coli* and enterococci concentrations were positively and significantly associated with HF183 presence (Wilcoxon rank sum, *p* < 0.05 for both).

### 3.8. Combinations for Introduction of Ruminant Contamination to Stored Water Samples

There were three combinations of household and human behavioral factors that resulted in the contamination of clean source water with ruminant feces during household storage. First, if ruminants were present in the household compound, safe water extraction methods were used, and water storage time was long (5 h or more). Second, if ruminants were present in the compound, unsafe water extraction methods were used, and there was no evidence of both soap and water at the household handwashing station. Third, if water storage time was long and there was no evidence of both soap and water at the household handwashing station.

**Figure 4:**
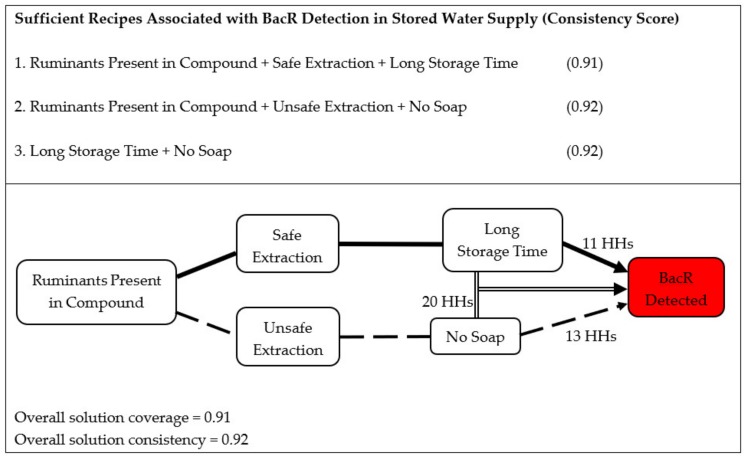
Combinations of causal conditions that are associated with the BacR molecular marker being detected in a household’s stored water supply, their consistency scores, and the number of households they explain. Each line type represents one combination; there are three in total: (i) ruminants + safe extraction + long storage time, (ii) ruminants + unsafe extraction + no soap, and (iii) long storage time + no soap. A household’s contamination may be explained by more than one combination.

Figure 4 illustrates these combinations and provides the consistency score for each. The overall coverage score of 0.91 relates to the fraction of households that have BacR contamination in their stored water supply and have conditions that fulfil at least one of the three combinations (26 of 28 households). In addition, the overall consistency score of 0.92 relates to the fraction of households with conditions that satisfy at least one of the three combinations and have BacR contamination in their stored water supply (26 of 28 households). For more details on score calculations, see Appendix B. A Tosmana diagram that shows the full solution space, the cases in each part of the space, as well as combinations associated with BacR contamination is shown in Figure S2.

## 4. Discussion

Microbial contamination was widespread in the water tested in this study. The majority of source and stored water samples collected in this study had *E. coli* and enterococci detected. This is higher than the WHO recommended levels of 0 CFU/100 mL for drinking water. We found evidence that ruminant fecal contamination was introduced post-collection from the water source. Nearly all stored water samples contained ruminant contamination (88%), whereas only 26% of the source water samples had ruminant feces detected. Ruminant feces can contain a number of zoonotic pathogens including *Campylobacter*, non-typhoidal *Salmonella*, *Cryptosporidium*, and *Toxoplasma gondii* [56,57,58], thus exposure to ruminant feces could present a health risk. Given that ruminant ownership was so common (~50% of households own a ruminant), and that their feces are used for household building material and fuel for fire in the study area, it is perhaps unsurprising that their feces often contaminate the water. However, exposure to ruminant feces as a transmission pathway for enteric illness is underexplored in the WASH field. Similar results regarding the high prevalence of ruminant contamination has been reported for households in rural and urban/peri-urban informal settlements in Bangladesh [12] and Tanzania [10]. In addition, Barnes et al. [59] showed an association between domestic animal presence/ownership and household drinking water contamination. Additional work should be done to assess the prevalence of zoonotic pathogens in waters contaminated by ruminant feces and how the persistence of the ruminant target varies relative to pathogen persistence in different environmental media (e.g., water and soil).

Although human contamination was positively associated with FIB contamination in drinking water collected within our study, human feces appears to be a secondary contributor of FIB contamination relative to ruminant feces. All study households reported access to a latrine. However, only 4% of those toilets fell into the category of “improved”, primarily because the rest lacked a concrete slab (i.e., surface that is easily cleanable) or were shared with multiple households. Despite this, only 4% (4 of 90) of the water samples contained the human-associated marker HF183 at concentrations above our detection limit of 50 copies per 100 mL (assuming a theoretical detection limit of 1 copy of target per qPCR reaction). The maximum concentration of the HF183 target detected in the water samples was 800 copies/100 mL, which, based on the range of concentrations of the HF183 target per culturable enterococci colony in human feces in the validation study, would correspond to between 80 and 800 CFU human fecal ENT per 100 mL of water. We found limited evidence of avian fecal contamination in drinking water (2% of samples) despite the majority of households owning chickens (76%). Prior work in rural Bangladesh, with similar animal ownership, found 10% of households with avian contamination in drinking water, compared to 0% of households with human contamination and 33% of households with ruminant contamination in water [12].

fsQCA yielded three distinct combinations of household and behavioral factors that led to ruminant contamination of clean source waters during storage in the home. The existence of three combinations suggests that there is no single way that stored water becomes contaminated with ruminant feces in the study population. The combinations revealed no necessary conditions. That is, none of the causal conditions are individually sufficient to result in contamination. This implies that multiple factors need to be collectively present for ruminant fecal contamination to occur. Ruminant presence in the compound was not a necessary condition for ruminant fecal contamination of a household’s stored water supplies as it was only included in two of the three combinations. Presumably, ruminant feces could be transferred into the household, for example, from a neighbor’s compound or due to widespread use of ruminant feces as building material. Somewhat counterintuitively, the use of ‘safe’ extraction methods in our study population was associated with ruminant fecal contamination of stored water if there were also ruminants in the compound and long water storage time. This suggests that safe extraction methods, defined to be when water is poured from the storage receptacle, may also introduce contamination during decanting, potentially due to biofilms on the storage container wall becoming dislodged or contamination along the rim of the container being washed along. Finally, the lack of both soap and water at the household handwashing station appeared as a factor in two of the three combinations. Evidence of soap served as an indicator for hand washing, thus lack of soap suggests reduced hand washing by the household. Handwashing with soap is especially critical in preventing food and water contamination [7] by ruminants [60], can reduce diarrheal disease risk by between 40% and 65% [61,62], and can protect against exposure to enteric zoonoses found in animal waste [63]. Although good hand hygiene is particularly protective at certain critical times [59], self-reported handwashing rates in this study population were only 20% before food preparation, 27% before eating, 20% before feeding children, 42% after cleaning children, and 76% after using the toilet.

The fsQCA methodology employed here offers benefits over average-effects-based analytical techniques (e.g., regression) to identify risk factors of fecal contamination in water. Striving to identify important behaviors or contextual factors that influence contamination transmission with average-effects-based techniques may lead to erroneous inference and misguided intervention design if there are in fact multiple combinations of contextual factors and behaviors that result in contamination. Often the failure of an intervention to produce the hypothesized impact is attributed to the intervention targeting the wrong pathway. However, it may be the case that the intervention successfully disrupts one pathway of contamination but fails to tackle other dominant pathways. No ‘necessary’ factors (i.e., a factor required for a case to have the outcome) were identified in the present study, highlighting the benefits of the fsQCA approach. The fsQCA methodology does not require *a priori* knowledge of these complex relationships and can identify multiple causal pathways that may lead to an outcome. Figure 4 identifies these pathways without providing insight on the sequence of events, if any, associated with contamination within these pathways.

There are several limitations of this work. The sampling scheme of this study means that the source water sample was collected at a later time than when the household collected their stored water, which was sampled. If a source exhibits temporal variation in quality, it could mean that the source water may have been of a different quality when the household visited as compared to when the enumerator visited. Water and fecal samples were also transported to the US for molecular analysis. Nucleic acid concentrations may decay during the transport process, although treatment with RNAlater was done to minimize decay. Nonetheless, since all samples were treated in the same way, there should be no systematic bias that would impact the comparison between households and between stored and source water. In addition, limited variation in values for indicators such as the use of dung in building their houses (in 43 of 45 households) and water treatment methods (43 of 45 households did not treat the tested stored water sample) meant that these indicators had to be excluded from the fsQCA analysis. Therefore, we were not able to evaluate these indicators as risk factors for ruminant contamination in stored water in our study. In addition, the quantitative sensitivity of the Avian GFD assay was very low (orders of magnitude lower than the sensitivity threshold), meaning that high concentrations of avian feces would need to be present in water in order for the target to be detected. Thus, avian contamination may be present in the water but not detected due to the limitations of the assay. Further work to develop a more sensitive avian assay is warranted, given increased concerns around poultry feces management in low-income country settings [56,64].

Our study provides evidence that ruminant contamination is widespread in household water supplies, and in many cases, despite safe storage and extraction practices. This highlights an underexplored health risk in the WASH field, given the potential for zoonotic disease transmission. Further work to understand the uses and handling practices of ruminant feces would yield insight to important exposure pathways, where water may just be one of many. This knowledge could inform the expansion of typical WASH interventions [65,66,67] to include safe contact with animals [59] and the safe disposal of animal waste [68,69]. Efforts to reduce exposure to ruminant feces would need to account for the fact that ruminants are also important nutritionally, financially, and culturally [70,71]. Conditions in this study are common throughout sub-Saharan Africa and Southeast Asia, where ruminant feces are used as a fuel or building material [72]. Understanding the sources and pathways of drinking water contamination remains a necessary step in reducing the risk of human exposure to fecal contamination.

## 5. Conclusions

Our study found evidence of post-supply contamination of drinking water with ruminant feces, highlighting the utility of molecular MST assays for understanding the extent of transmission of species-specific fecal contamination in household environments. Given the number of zoonotic pathogens that can be found in ruminant feces, its presence in drinking water implies a public health risk. Further research to understand the extent of zoonotic pathogen carriage in animals in different environmental contexts is warranted to draw robust public health conclusions from contamination data. Studies to assess the persistence of the molecular targets in different environmental media compared to zoonotic pathogens would also help more precisely define the health risks associated with target detection.

fsQCA was used to identify three combinations of causal conditions associated with the introduction of ruminant fecal contamination to household drinking water after collection from a source, with no necessary or sufficient factors being identified. Of particular note was that ruminant presence in the household was not a necessary condition and that safe extraction methods was a causal condition in one contamination pathway. This suggests that ruminant feces are widespread in the study area and methods perceived to be safe are insufficient to prevent contamination. This study represents a novel application of fsQCA and reveals limitations of standard regression techniques for understanding complex phenomena such as the various transmission pathways of fecal contamination in household environments. Additional studies to understand household behaviors and practices related to animal feces will help inform effective and culturally appropriate feces and water management strategies.

## Figures and Tables

**Table 1:** Measurement Table and Summary of Indicators for fsQCA.

Construct	Definition	Theoretical Relevance	Hypothesized Effect	Indicator & Coding Scheme
Ruminant contamination in stored water supply given uncontaminated source water	The extent of fecal contamination from ruminants (cattle, goats, and sheep) in the household’s main stored drinking water supply.	Outcome variable	Outcome variable	Indicator: Detection of BacR molecular marker in a 100mL sample of the household’s stored water Coding: 1 = BacR MST molecular marker detected, 0 = Otherwise
Proximity of ruminant feces production to stored water	Presence of ruminants and/or their feces within compound of household from which stored water sample was taken	Ruminant presence in the compound makes feces in the environment likely and be difficult to keep out of stored water supply due to proximity.	Positively associated with likelihood of contamination	Indicator: Self-reported number of ruminants living in the compound Coding: 1 = Some ruminants live in household compound 0 = No ruminants live in household compound
Opportunity for introduction of contamination into stored water	Length of time water is stored, which may allow ruminant fecal contamination to enter stored drinking water supply	The longer the time that water is in storage before use, the greater the chance that contaminated objects (e.g., hands, utensils etc.) will be introduced into the water	Positively associated with likelihood of contamination	Indicator: The number of hours that have elapsed since currently stored water was fetched from the source, as reported by the respondent. Coding *: 0.95 = Water self-reported as collected 1 day ago 0.5 = Water self-reported as collected 4.5 h ago 0.05 = Water self-reported as collected 1 h ago * These values were provided as calibration points for the fsQCA software to score a range of storage times from 1 h to 2 weeks.
Unsafe water extraction method	The extent to which a household’s method for extracting water from storage is likely to result in contamination of the water, e.g., by hands and other contaminated objects	Retrieving water using hands and other potentially contaminated objects allows for the (re)contamination of the stored water supply.	Positively associated with likelihood of contamination	Indicator: Observed method of water retrieval when respondent asked to get a cup of water for child (or themself, if no child) Coding: 1 = Respondent inserted hands or an object into the water; 0 = Respondent poured water from the top of the storage container
Lack of supportive household environment for handwashing	The extent to which household members lack regular access to supplies essential for good hand hygiene	The absence of soap is hypothesized to increase the likelihood of BacR contamination in the stored water supply	Positively associated with likelihood of contamination	Indicator: Observed presence of water and soap together at the home handwashing station 1: Water and soap not at home handwashing station when enumerators visited 0: Water and soap at home handwashing station when enumerators visited

**Table 2:** Sensitivity and specificity of the MST source-specific assays as concluded from the binary analysis, and whether assay is deemed sensitive or specific based on binary and quantitative metrics.

Assay	Binary Analysis		Binary Metric		Quantitative Metric	
	Sensitivity	Specificity	Sensitivity	Specificity	Sensitivity	Specificity
HumM2	100%	60%	yes	no	no	no
HF183	100%	48%	yes	no	no	yes
BacHum	80%	44%	yes	no	no	no
Rum2Bac	100%	67%	yes	no	yes	yes
BacR	100%	87%	yes	yes	yes	yes
BacCow	100%	60%	yes	no	yes	no
Avian GFD	100%	48%	yes	no	no	yes

**Table 3:** Household characteristics (*n* = 45).

	Metric	Average (Range)
	Household (HH) size	5 (2–10)
	Formal Education, mother	Primary education completed (none to post-secondary)
Assets	Household has Electricity	9% (4 HHs)
	Bicycle Ownership	42% (19HHs)
	Mobile Phone Ownership	76% (34 HHs)
Water Source	Spring	84% (38 HHs)
	Well	11.1% (5 HHs)
	Stream	2% (1 HH)
	Borehole	2% (1 HH)
Toilet	Access to toilet facility	100% (45 HHs)
	Private Use	47% (21 HHs)
	For shared, mean number of households sharing	3.2 HHs
Animal Ownership	Cattle	47% (21 HHs)
	Goats	4% (2 HHs)
	Sheep	7% (3 HHs)
	Poultry	76% (34 HHs)
	Dogs	31% (14 HHs)
	Cats	9% (4HHs)
	HH Water Treatment	13% (6 HHs)

**Table 4:** MST molecular marker detection in paired source and stored water samples (*n* = 45 households). Households returning the same result for both samples have concordant pairs, whereas those with only one of their water samples showing contamination have discordant pairs.

Assay	Both Source and Stored Water Contaminated	Both Source and Stored Water Uncontaminated	Only Source Water Contaminated	Only Stored Water Contaminated
BacR	11 (24%)	4 (9%)	1 (2%)	29 (64%)
HF183	1 (2%)	42 (93%)	2 (4%)	0 (0%)
Avian GFD	0 (0%)	43 (96%)	0 (0%)	2 (4%)

**Figure A1:**
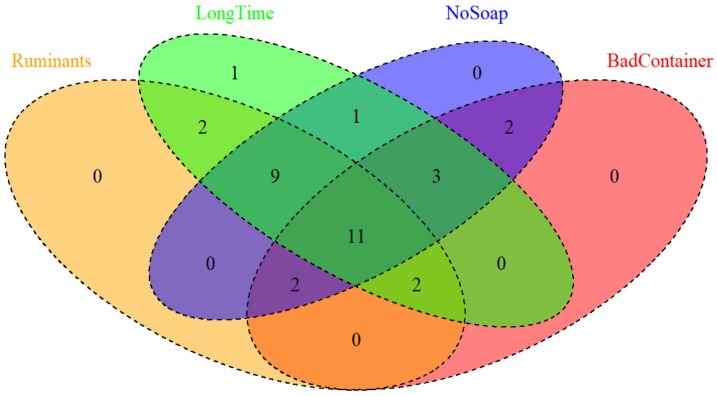
Venn diagram showing the number of households who have various combinations of the causal conditions chosen.

**Figure A2:**
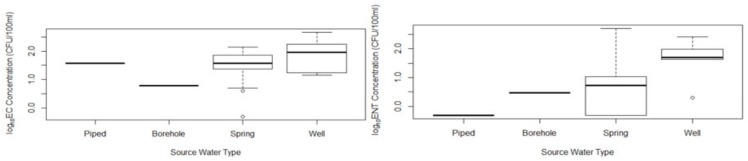
Boxplots of the log_10_-transformed concentrations of *E. coli* (**left**) and *Enterococci* (**right**) in household source water samples, stratified by type of source. The midline of the box represents the median of the data, with the upper and lower bounds of the box showing the first and third quartile. The whiskers show the extremes of the data that is within 1.5 times the interquartile range. Any data points outside this range are plotted as outlier circles.

## Supplementary Materials

The following are available online at https://www.mdpi.com/1660-4601/17/2/608/s1. Table S1: Number and types of fecal samples collected, Figure S1: Master curve plots for the Avian GFD, BacR and HF183 assays generated from qPCR runs, Table S2: Methods for MST assays used in this study, Figure S2: Tosmana diagram showing the number of households that have each combination of the causal conditions and their consistencies with the outcome condition.

## Appendix A

To assess inhibition, a modified ‘spike and dilute’ method was used [46,73]. One fecal composite per animal source type as well as five randomly chosen water samples were processed to test for inhibition for each MST assay. Both the fecal composites and water samples were processed at two different 10-fold dilution levels, and each dilution was spiked with a different concentration of standard. For instance, the water, chicken, duck, dog, and cow samples were processed undiluted as well as at a 1:10 dilution. The undiluted sample was spiked with 10^4^ copies per μL standard and the 1:10 diluted sample was spiked with 10^3^ copies per μL standard.

If, after the DNA extraction process, a sample had a DNA concentration in the eluent greater than 100 ng DNA/μL, the DNA concentration in the sample was considered too high to be processed undiluted. In these instances, the two different ten-fold dilution levels instead became 1:10 and 1:100. For the ruminant-specific assays, DNA concentrations in the eluent of the ruminant fecal samples (i.e., cow, goat, and sheep) warranted further dilution to a third (1:1000) dilution level. Thus, the ruminant samples were processed at 1:10, 1:100, and 1:1000 dilutions for the ruminant assays, and spiked with 10^4^, 10^3^ and 10^2^ copies per μL standard respectively. All samples tested for inhibition were processed in duplicate. If the difference in mean C_T_ values between the two dilutions was greater than 2, the more concentrated sample tested was considered uninhibited [46].

## Appendix B

Calibration of the indicator for Long Storage Time was conducted in the software with the following equation:

$$Target\;Variable\;LongStorageTime=calibrate\left(TimeCol,24,4.5,1\right)$$

where TimeCol is the respondent’s self-reported survey response of how long ago their current household stored water supply was collected.

After all conditions, causal and outcome, are scored, a truth table is generated using the Truth Table Algorithm function under the Analyze tab such that cases are aggregated based on the scores of their causal conditions, as shown in Table A1. For ease of inspection, each causal condition is scored by the software in a binary fashion in the truth table even if it is otherwise coded fuzzy in the analysis; i.e., all scores above 0.5 are shown as 1, all scores below 0.5 shown as 0, with no scores of exactly 0.5 permitted. Scores that are not Boolean impact the raw consistency metrics. For example, if there are two households with scores of 1 across all causal conditions as well as the outcome, the consistency score would be 1. However, if these households had differed in the outcome, with one showing contamination and the other not, the consistency score would be 0.5. If both households showed contamination and were identical in all other respects bar having one causal condition that was fuzzy, the consistency score would be close to but not 1.

**Table A1:** Truth table showing the number of households who have various combinations of the causal conditions chosen.

Ruminants	Unsafe Extraction	Long Storage Time	No Soap	Number	Raw Consist.	PRI Consist.	SYM Consist.
1	1	1	1	11 (33%)	0.943434	0.943434	0.943434
1	0	1	1	9 (60%)	0.885057	0.885057	0.885057
0	1	1	1	3 (69%)	0.935058	0.935058	0.935058
1	0	1	0	2 (75%)	1	1	1
1	1	1	0	2 (81%)	0.5	0.5	0.5
0	1	0	1	2 (87%)	0.438849	0.438849	0.438849
1	1	0	1	2 (93%)	0.858064	0.858064	0.858065
0	0	1	0	1 (97%)	0	0	0
0	0	1	1	1 (100%)	1	1	1
0	0	0	0	0 (100%)			
1	0	0	0	0 (100%)			
0	1	0	0	0 (100%)			
1	1	0	0	0 (100%)			
0	1	1	0	0 (100%)			
0	0	0	1	0 (100%)			
1	0	0	1	0 (100%)			

The Venn diagram in Figure A1 helps better visualize the full range of combinations of causal conditions described by the households in this study, as well as the number of cases there are for each combination. However, it does not show the number of cases within each combination of causal conditions that are positive or negative with respect to the outcome.

Two elements of further input are required to code the outcome variable for each configuration of causal conditions represented by the household data before combinations associated with the outcome condition can be determined. This is because there may be multiple cases that have the same configuration of causal conditions but different outcomes. However, the software requires that each configuration has a definitive outcome, positive or negative, regardless of the variation between cases. The first element of input is the minimum number of cases (households) that are required before a given set of causal conditions in the truth table is included in the process of deriving combinations that are associated with BacR contamination. This was set to 1 in this analysis. The second element of input is the minimum raw consistency score necessary to set the outcome condition for that combination of causal conditions to 1. A widely accepted threshold is 0.8 [54], which was also adopted here. Establishing these thresholds via the Delete and Code function under the Edit tab of the Edit Truth Table window enables the software to compute the various combinations associated with the outcome condition.

These inputs allow the analyst to continue with the analysis to determine combinations of causal conditions associated with the outcome. When conducting Standard Analyses (the analysis tool in the software used to compute combinations without placing further constraints on the solution such as minimizing positive cases, negative cases, don’t care cases, and remainders), each causal condition was listed such that ‘they should contribute to BacR when cause is present’ because this is how the coding scheme was set up given their hypothesized effects. (In situations where the hypothesized effect is unknown, the ambiguous option of ‘present or absent’ can be chosen, as can the option of ‘absent’ when the hypothesized effect is associated with the absence of a causal condition.)

To assess soap as a proxy measure, sensitivity analyses were conducted, where poverty and a lack of education were added as causal conditions in the fsQCA analysis initially separately and then together. The combinations of causal conditions did not substantively change for any of the situations: When poverty was added independently, it only changed one combination by replacing the presence of ruminants, itself an indicator that is often linked to higher socioeconomic status. When a lack education was added independently, it appeared in a combination with a lack of soap and unsafe extraction methods, suggesting that a lack of education may also be reflected in having fewer resources. Including both a lack of education and poverty resulted in the same combinations as simply including poverty alone. As such, it was not deemed necessary to include separate indicators for poverty and a lack of education in the analysis.

## Appendix C

There was no statistical association shown from bivariate tests between the self-reported presence of avian species or ruminants in the compound at the time water was collected and the associated MST molecular marker in household stored water samples (*p* = 1 for Avian-GFD; *p* = 0.58 for BacR). 31 of the 34 households (91%) that had ruminants in the compound had BacR contamination detected in their stored water sample, whilst 9 of the 11 households (82%) that did not detected similar contamination. On the other hand, 37 of the 39 households that owned poultry (95%) did not have Avian GFD in their stored water sample, whilst all 6 households that did not showed the same contamination. These data are reported in Table A2 and Table A3.

**Table A2:** Self-reported presence of avian species with detection of Avian GFD molecular marker in stored water.

	Avians Present	Avians Absent
Avian GFD Detected	2	0
Avian GFD Not Detected	37	6

**Table A3:** Self-reported presence of ruminants with detection of BacR molecular marker.

	Ruminants Present	Ruminants Absent
BacR Detected	31	9
BacR Not Detected	3	2

## Appendix D

As shown in Figure A2, the quality of source water as evaluated by FIB metrics varied by source type, with quality broadly worsening in the following order: piped/borehole, spring, well. The order differed mildly depending on whether the water was evaluated for *E. coli* or *enterococci*. Given this variation, the same source and stored water analysis was conducted for the subset of households whose source was a spring since springs were the most common water source. However, the result and its significance were unchanged: there were significantly higher levels of *enterococci* but no *E. coli* in stored relative to source water.
